# Supplementary material for: Quantifying the intra- and inter-species community interactions in microbiomes by dynamic covariance mapping
Source: Nat Commun. 2025 Jul 9;16:6314. doi: 10.1038/s41467-025-61368-y (PMC12238654; doi:10.1038/s41467-025-61368-y)
Supplement: Supplementary file 1 — Supplementary Information [file 41467_2025_61368_MOESM1_ESM.pdf]

Supplementary Information for

"Quantifying the intra- and inter-species community interactions in microbiomes by dynamic covariance mapping" by Gencel *et al.*

Correspondence: adrian.serohijos@umontreal.ca

CONTENTS:

Supplementary Figures

Supplementary Fig. 1 DCM captures the interaction strength matrix and defines dynamical domains in Lotka-Volterra systems. 3
Supplementary Fig. 2 Application of DCM to a community perturbed by pathogenic bacteria........ 5
Supplementary Fig. 3 Barcode dynamics in im, rm, and gf cohorts. 6
Supplementary Fig. 4 Number of input genomes for barcode PCR amplification. 7
Supplementary Fig. 5 Barcode and community diversity dynamics in im, rm, and gf cohorts. 8
Supplementary Fig. 6 Determining the number of dominant clonal lineages. 9
Supplementary Fig. 7 Dynamic covariance mapping on the gf cohort defines distinct phases of colonization. 11
Supplementary Fig. 8 Dynamic covariance mapping analysis on the im and nc cohort defines distinct phases of colonization. 12
Supplementary Fig. 9 Principal components of the eigenvalue matrix of over the entire time-series. 13
Supplementary Fig. 10 Growth rates of clones screened for whole-genome sequencing. 14
Supplementary Fig. 11 Effect of genome quantity and PCR bias on lineage cluster clones (skewed initial distribution) 16
Supplementary Fig. 12 Effect of genome quantity and PCR bias on lineage cluster clones (uniform initial distribution) 18
Supplementary Fig. 13 Three-species Lotka-Volterra system with stable oscillatory coexistence. 21
Supplementary Fig. 14 Schema for shaped-based co-clustering between *E. coli* clone and community dynamics. 22
Supplementary Fig. 15 Comparison of rm cohort co-clustering using shape-based distance (SBD) with lag-penalized weighted clustering (LPWC). 24

Supplementary Text

A. Sensitivity of dominant clonal lineage clusters on experimental parameters 15
A.1. Effect of number of input genomes to PCR amplification of barcode region 15
A.2. Effect of PCR jackpotting 17
A.3. Effect of skewedness of barcode frequency at time t=0 17
A.4. Plasmid vs. chromosomal barcoding 19
B. DCM analysis on generalized Lotka-Volterra models and the effect of time-series sampling accuracy 20

B.1. Correspondence with generalized Lotka-Volterra 20
B.2 Capturing community shifts without prior knowledge using gLV 20
B.3. Effect of time sampling on community abundances 21
C. Co-clustering between E. coli clonal cluster lineages and the community dynamics 21
C.1. Assessing the biological significance of the co-clustering of clonal clusters and community dynamics. 22
C.2. Robustness to E. coli clonal cluster lineages and the community dynamics to choice of distance and clustering methods 23

Supplementary Figures

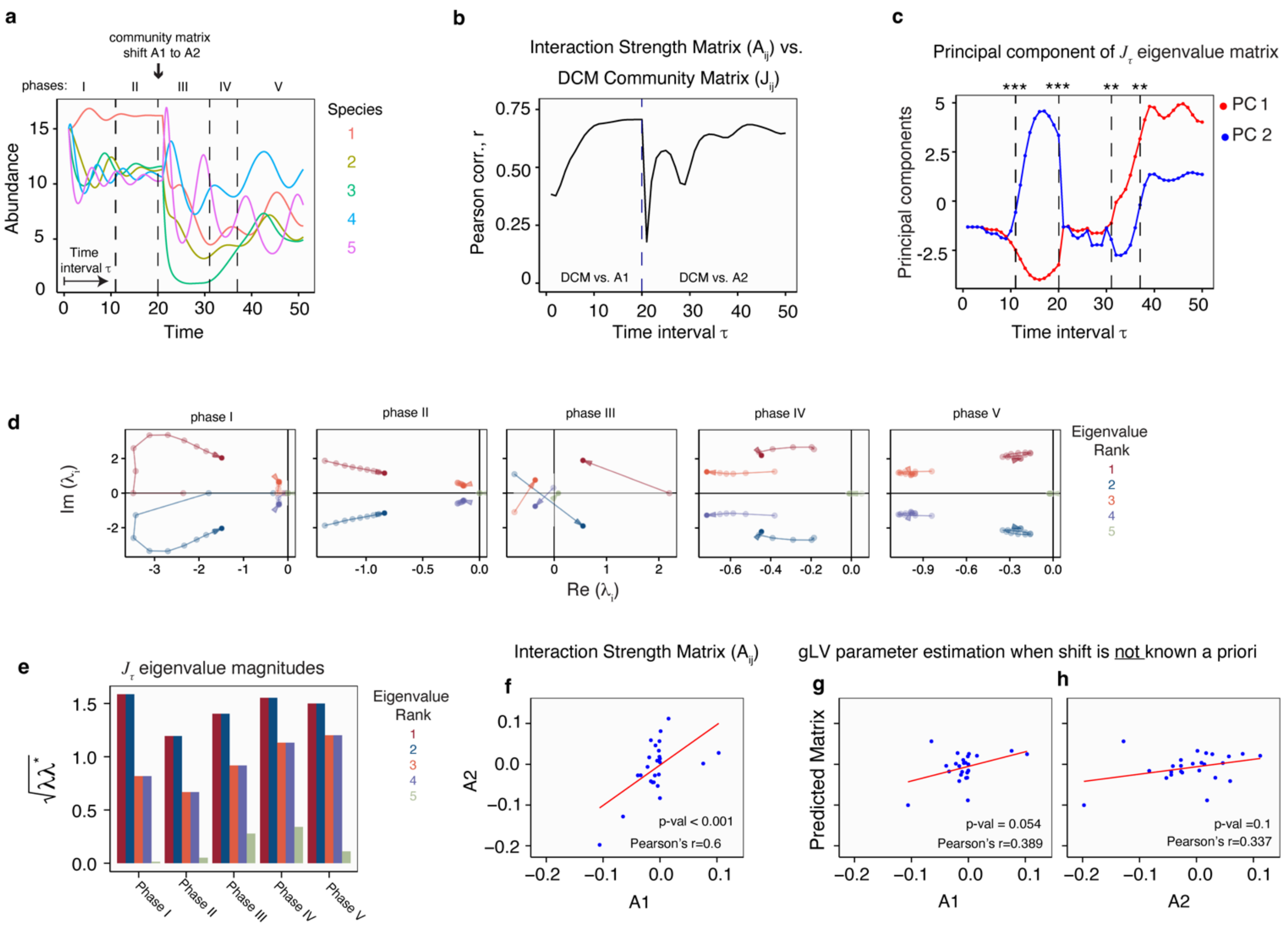

Supplementary Fig. 1 DCM captures the interaction strength matrix and defines dynamical domains in Lotka-Volterra systems.

a, An illustrative example of DCM analysis on a 5-species gLV system with an interaction strength matrix A1 that shifts to matrix A2 at time $t = 20$. The correlation between elements of matrices A1 and A2 is in panel f. The species abundance time series are the solution to gLV and all species abundances are initialized to 15 at $t = 0$. **b**, Correlation between the Community matrix estimated by DCM's J_{ij} and A1 matrix in the interval $t = [0,20]$ and between J_{ij} and A2 matrix in the interval $t = [0,20]$. The time-dependent J_{ij} reflects the shift in the 5-species interaction of the Lotka-Volterra matrix. **c,** Principal components (PC1 and PC2) of the eigenvalue matrix over the time interval for the 5-species community shown in panel **a**. A change in the direction of either PC1 or PC2 is indicative of a dynamical shift in the community. This shift is detected using change-point analysis applied to the PC1 and PC2 timeseries (main Text). Five distinct phases are identified based on the matrix eigenvalues and their dynamic interpretations (stable/unstable or oscillatory (panel d)). The stars denote confidence level of identifying a dynamical shift: *** is highly confident phase boundary and ** is intermediate confidence (Methods). One of the major dynamic temporal boundaries corresponds to the shift in ecological matrix A1 and A2 (between phases II and III). Other phase boundaries correspond to approach to equilibration of the community from an initial set of abundances (panel d). **d-e,** The eigenvalue colors correspond to the rank of their magnitude (panel **e**) (See also Supplementary Movie 1). Phase I is the transient approach to equilibrium of the abundances initially set to 15, followed by their equilibrium as dictated by matrix A1

(phase II). Phase III is the sudden change in abundances, and destabilization (real primary eigenvalues), due to ecological shift from A1 to A2. Phase IV is the approach to new equilibrium abundances dictated by A2, and Phase V is the steady-state equilibrium defined by the new interaction matrix. **f**, Correlation between the pre- and post-perturbation interaction matrices A1 and A2, respectively. **g–h**, Comparison of interaction matrices estimated from the time series data in panel a using a gLV model with a constant interaction matrix (that is, agnostic to the community shift) (Supplementary Information B2). In the first panel, the gLV predicted interaction matrix is compared to A1, yielding a Pearson correlation coefficient of $r = 0.39$ (P-value=0.054). The second panel compares the predicted matrix to A2, where the correlation is slightly lower ($r = 0.34$, P-value = 0.1)

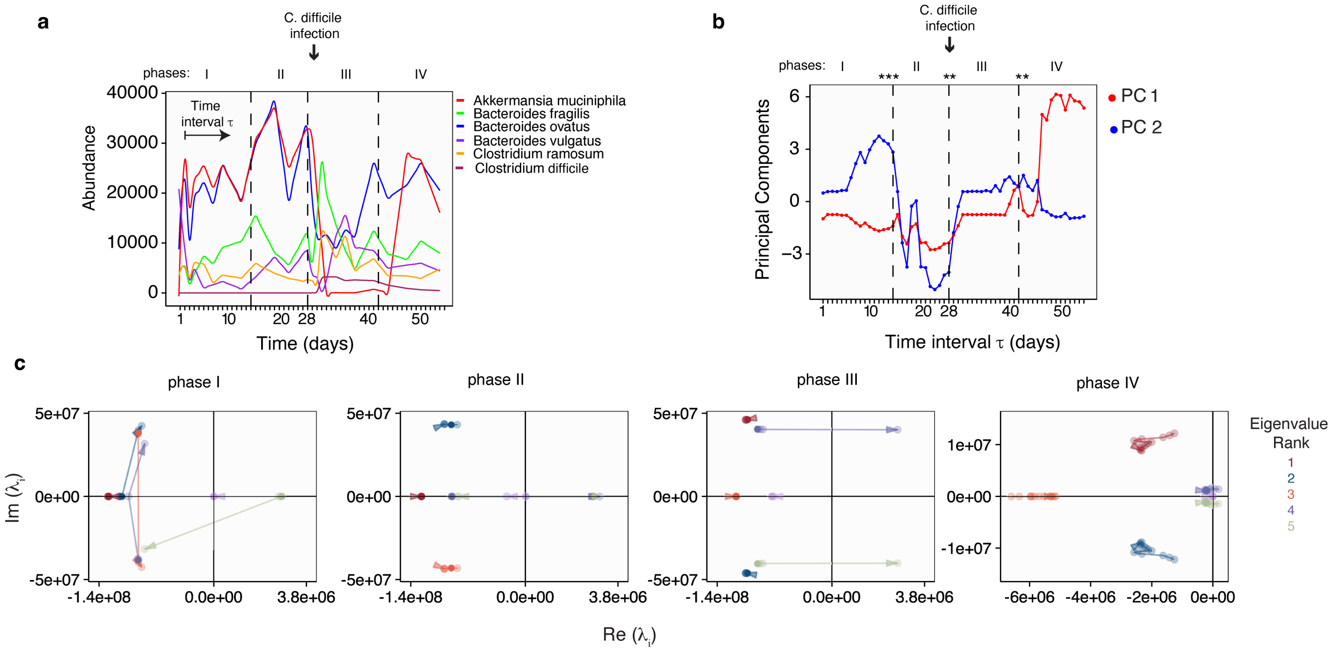

Supplementary Fig. 2 Application of DCM to a community perturbed by pathogenic bacteria.

a, Bucci et al.[1] used gnotobiotic mice pre-colonized with a Gnotocomplex microflora for 28 days, infected the mice with *C. difficile* at day 28, and then monitored the microbiome for another 28 days. Abundance timeseries shows the microbiome's most abundant five species during the 56-day experiment. **b & c**, Principal components PC1 and PC2 of the eigenvalues of the Jacobian. Change-point analysis identifies four phases. Phase I reflects the entry and establishment of the Gnotocomplex microflora, while Phase II reflect the transient instability detected from the rise in *A. mucinphilia* and *B. ovatus*. Phase III is the collapse in abundance upon entry of *C. difficile*. Phase IV is the return to stability accompanied by the increase of *A. mucinphilia* and *B. ovatus* to baseline levels in Phase I. See also Supplementary Movie 2.

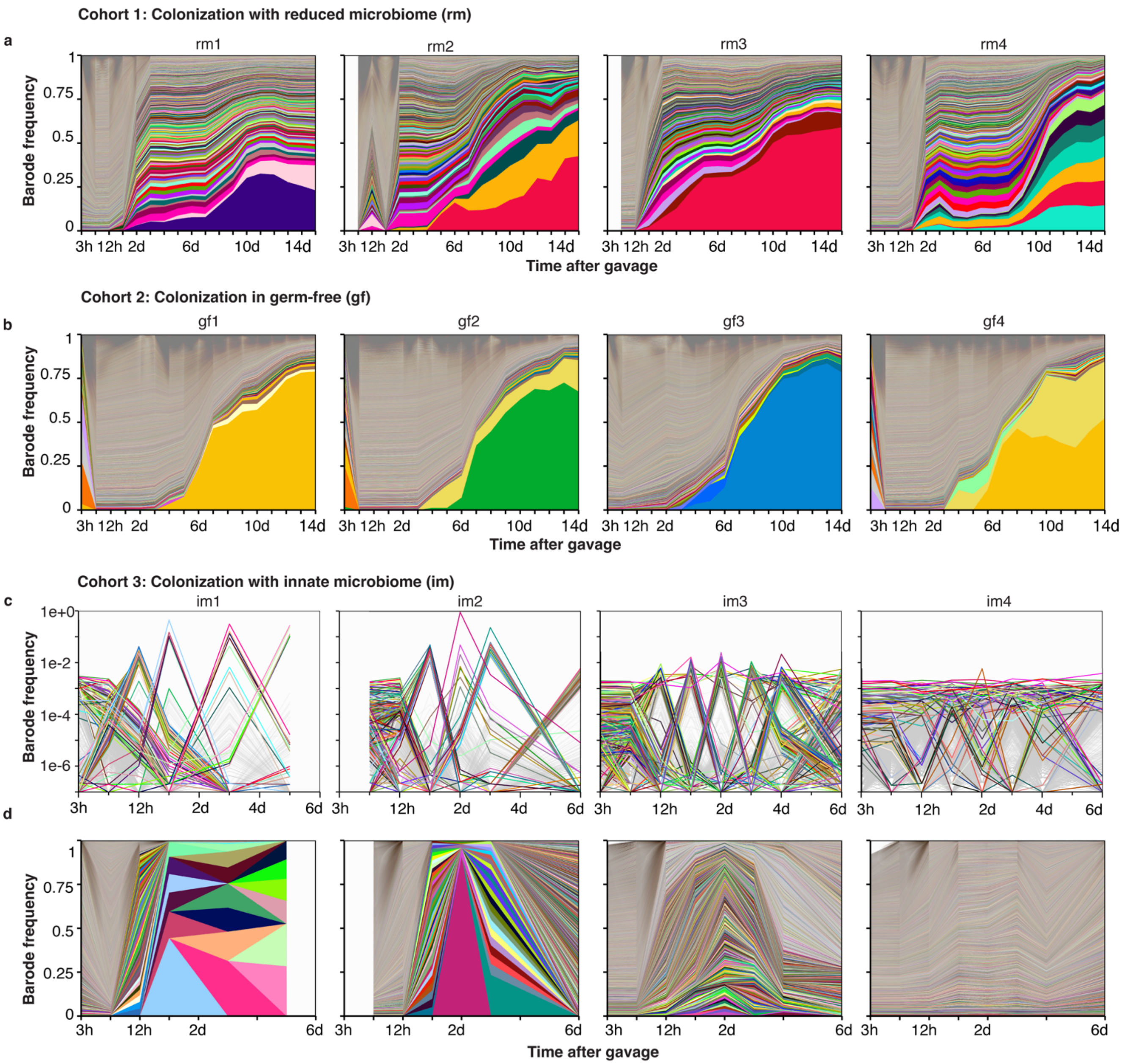

Supplementary Fig. 3 Barcode dynamics in im, rm, and gf cohorts.

a-b, Barcode dynamics of cohorts 1 and cohort 2 in linear scale. Each column corresponds to replicate mouse 1 to 4. The color corresponds to Fig. 2a for cohort 1 and 2d for cohort 2. **c-d**, Barcode dynamics for cohort 3 in log-scale (panel c) and linear scale (panel d). Colors in both panels correspond to the same barcodes.

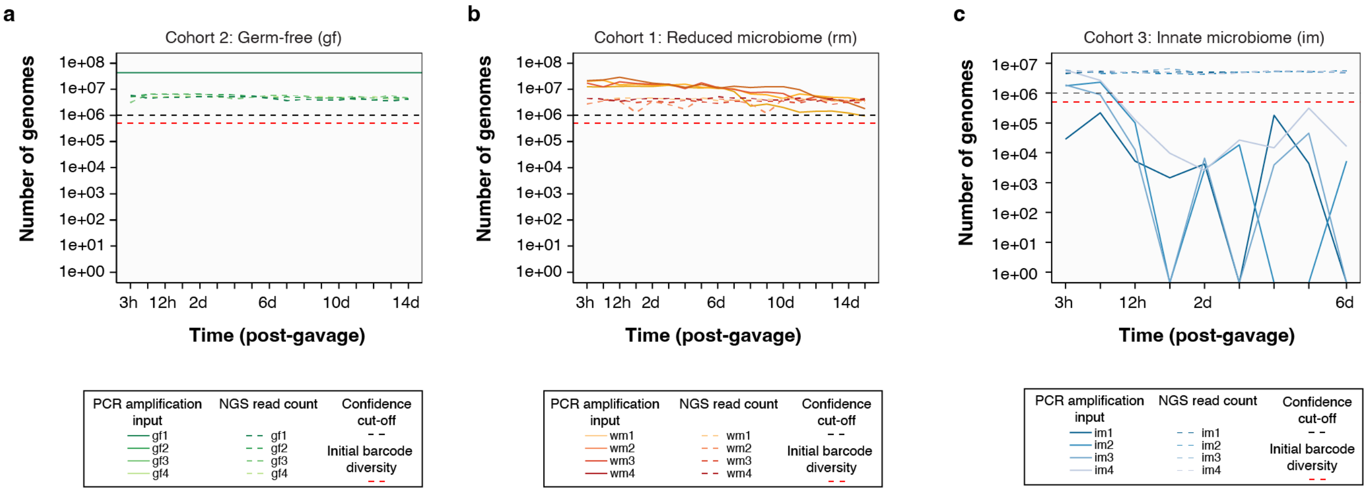

Supplementary Fig. 4 Number of input genomes for barcode PCR amplification.

a, The number of input genomes for PCR amplification is calculated as follows: No. of *E. coli* genomes = (200 ng of community gDNA) × (fraction of *E. coli* from 16S) × (1 genome/0.0046 pg of DNA), where the weight of an *E. coli* genome is 0.0046 pg (Bionumbers[2]). For the germ-free cohort, the fraction of *E. coli* in the community is, by definition, 100%. We also count the number of reads from next-generation sequencing. Accounting for potential dropout in barcode due to the efficiency of genome extraction and potential PCR bias ("jackpotting"), we set the confidence cut-off for the number of input genomes at ~1e+6 (dashed black line; see also detailed calculation and analysis in the Supporting Information). The initial barcode diversity, ~500,000, is indicated by a dashed red line. **b,** Similar to panel a, but for the reduced microbiota cohort. **c,** Similar to panel a, but for the colonization with innate microbiota. Unsuccessful colonization (see Fig. 1a) implies that the number of *E. coli* genomes is below our confidence cut-off, making it prone to sampling bias.

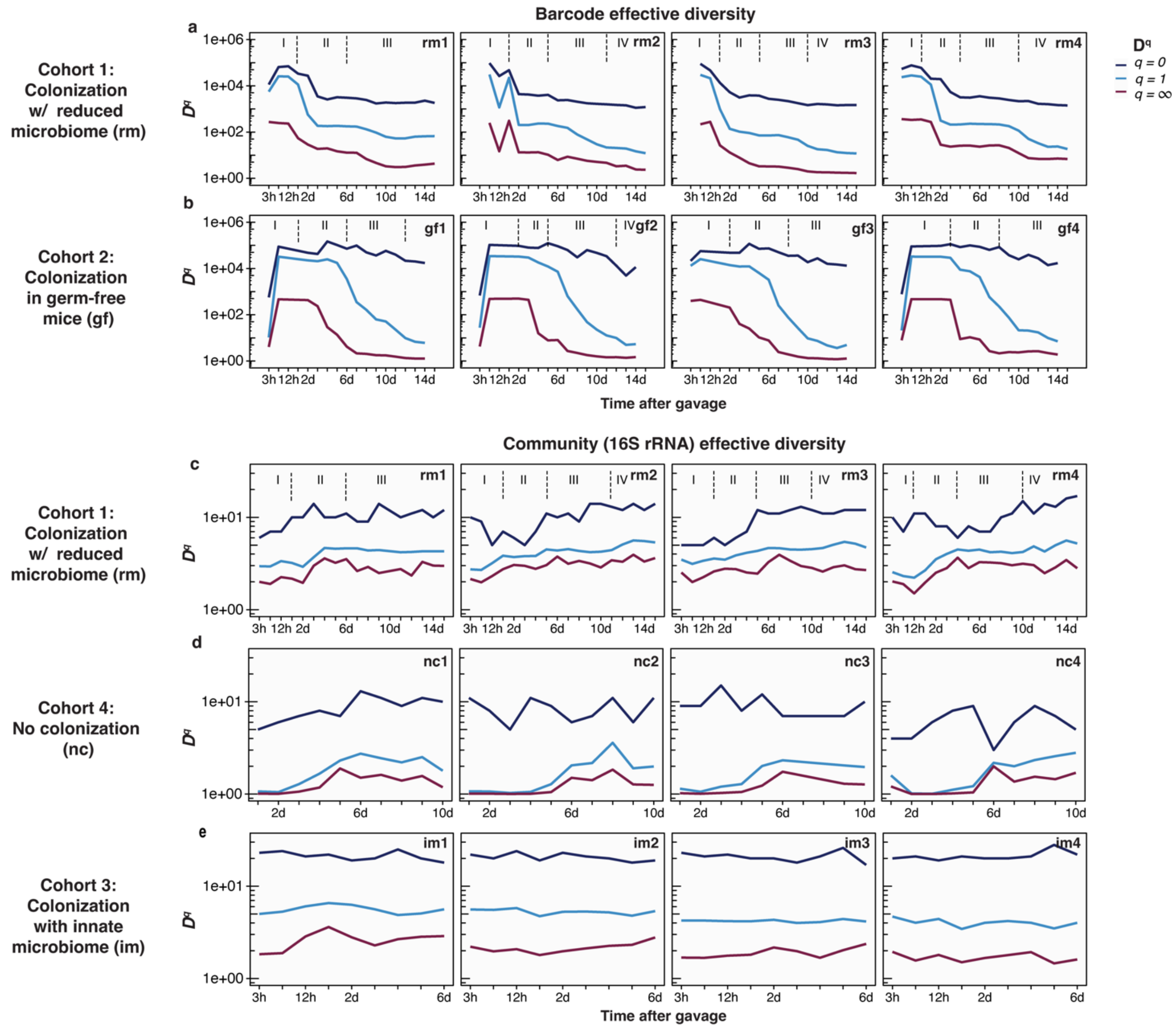

Supplementary Fig. 5 Barcode and community diversity dynamics in im, rm, and gf cohorts.

a-b, Effective diversity index of *E. coli* chromosomal barcodes, where is the frequency of barcode , is the total barcode count, and is the order of the diversity. Effective diversity reports the count of unique barcodes (), frequency-weighted diversity (), or inverse frequency of the dominant barcode (). The phases correspond are those identified by DCM. **c-e**, Effective diversity for the microbiota based on the frequency of bacterial families from 16S rRNA profiling.

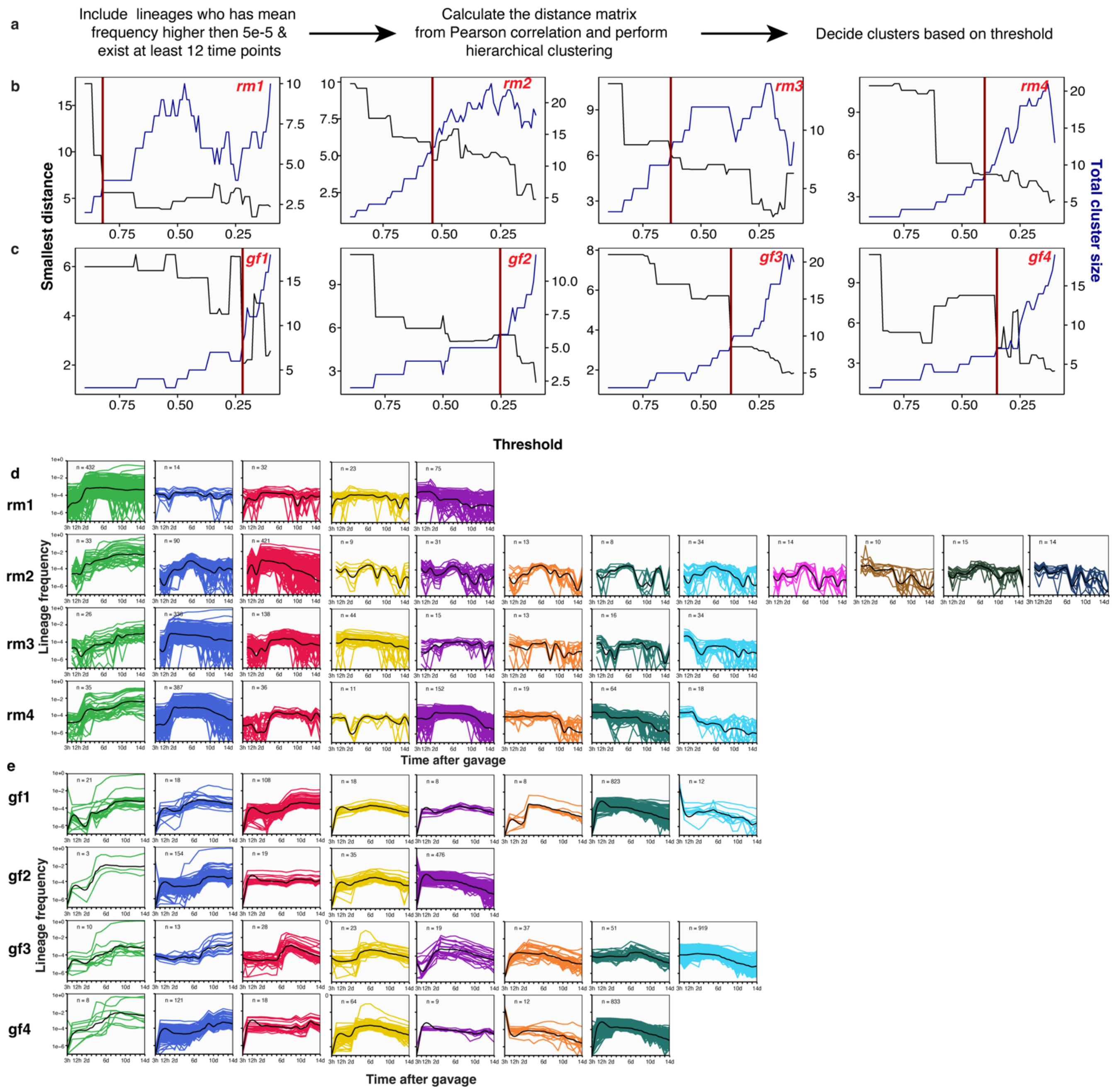

Supplementary Fig. 6 Determining the number of dominant clonal lineages.

a, The pipeline for estimation of putative clonal clusters from the frequency time series of the chromosomal barcodes (extended description in the Methods). **b-c,** A specific value for the threshold distance (Pearson correlation) in the hierarchical clustering defines a total number of clusters (blue curve) as well as a distance between the most similar clonal clusters ("Smallest distance", black dots). When the threshold is small there are many clusters, but some are similar to each other. Conversely, when the threshold is large, there are too few clusters, where even barcodes that do not have similar time series are grouped together (Methods). In practice, the cut-off is chosen to be the cross-over between the smallest distance between cluster centroids (our loess average) and the number of clusters. The chosen cut-off for each mice is indicated by the red curve. **d,** Dominant clusters for the mice with reduced microbiota (rm). The colors correspond to Figure 2b. Colored lines correspond to unique chromosomal barcodes in the cluster. Black lines correspond to the LOESS average. The number of unique raw barcodes that belong to the cluster is indicated. The clonal lineage clusters (or simply

“clonal clusters”) are ordered, starting from the left, based on their average barcode frequency on the last day. **e**, Dominant clusters for the germ-free mice (gf). The colors correspond to Figure 2e.

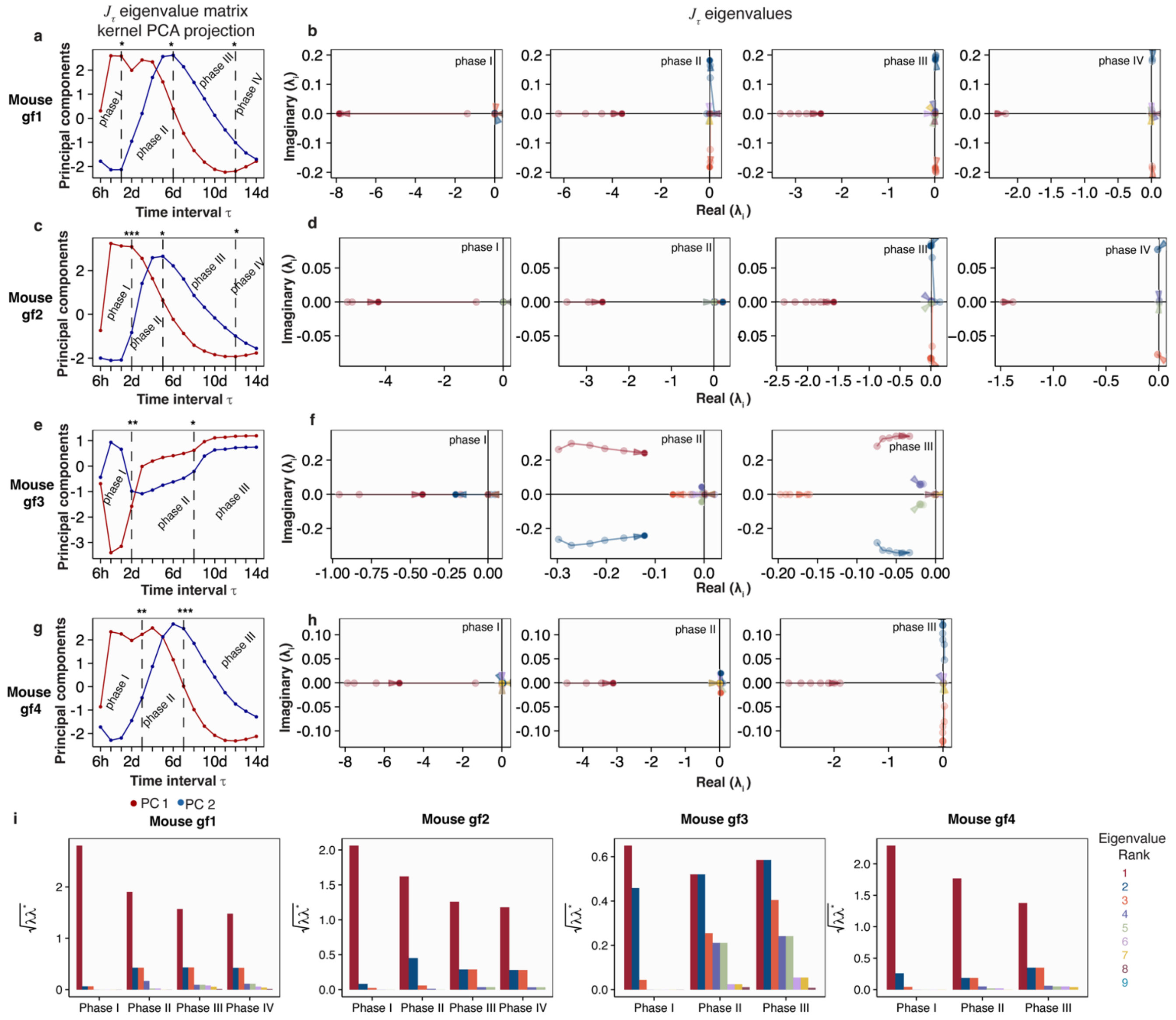

Supplementary Fig. 7 Dynamic covariance mapping on the gf cohort defines distinct phases of colonization.

a-b, Principal components (PC1 and PC2) of the eigenvalue matrix of over the time interval for mouse rm1. A change in the direction of either PC1 or PC2 is indicative of a dynamical change in the community. Using this criterion, three distinct phases are identified based on the eigenvalues of and their dynamic interpretation stable/unstable or oscillatory (panel b). The eigenvalue colors correspond to the rank of their magnitude (panel i). Phase I is the transit of the E. coli through the gut. Phase II is the beginning of the rise of dominant clone. Phase III is after the clonal sweep of the dominant clone. **c-d**, PCs and phases for mouse gf2, which has similar behaviour as gf1. **e-f**, PCs and phases for gf3. It is distinct from gf1, 2, and 4 due to a much faster transit time whereby almost all barcodes had transited by 3h, which is our first sampling. **g-h**, PCs and phases for gf4. **i**, The magnitude of the eigenvalues.

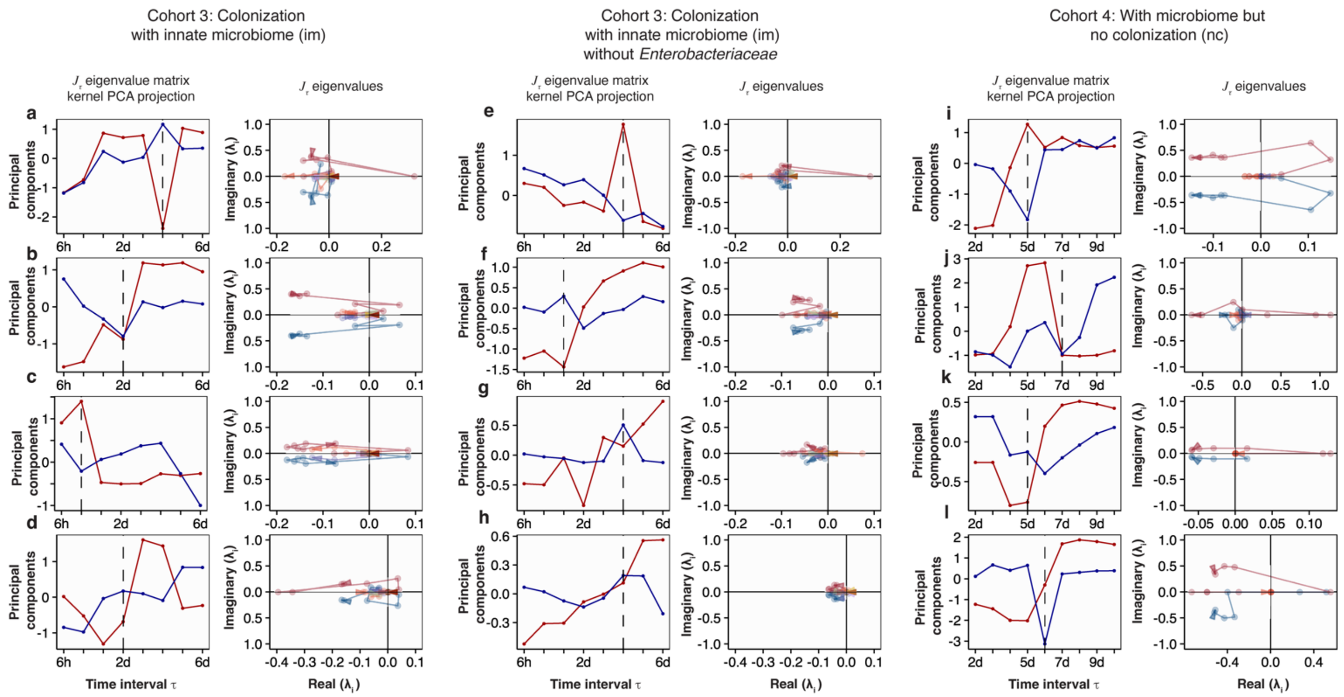

Supplementary Fig. 8 Dynamic covariance mapping analysis on the im and nc cohort defines distinct phases of colonization.

a-d, Principal components (PC1 and PC2) of the eigenvalue matrix of over the time interval for the im cohort. We did not observe distinct phases that were reproducible across mice cohorts. Explored eigenvalues over the time-series are shown on the right panel. See also Supplementary Movies 11-14. **e-h**, DCM analysis of the im cohort when *Enterobacteriaceae,* colonizing E. coli's family, is excluded. Removing the colonizer reduces the imaginary components of the eigenvalues. See also Supplementary Movies 15-18. **i-l** , PC1 and PC2 of the eigenvalue matrix of over the time interval for cohort nc. Employing dynamic change criteria, we identified two distinct phases that correlate with the microbial community's recovery following antibiotic treatment. See also Supplementary Movies 19-22.

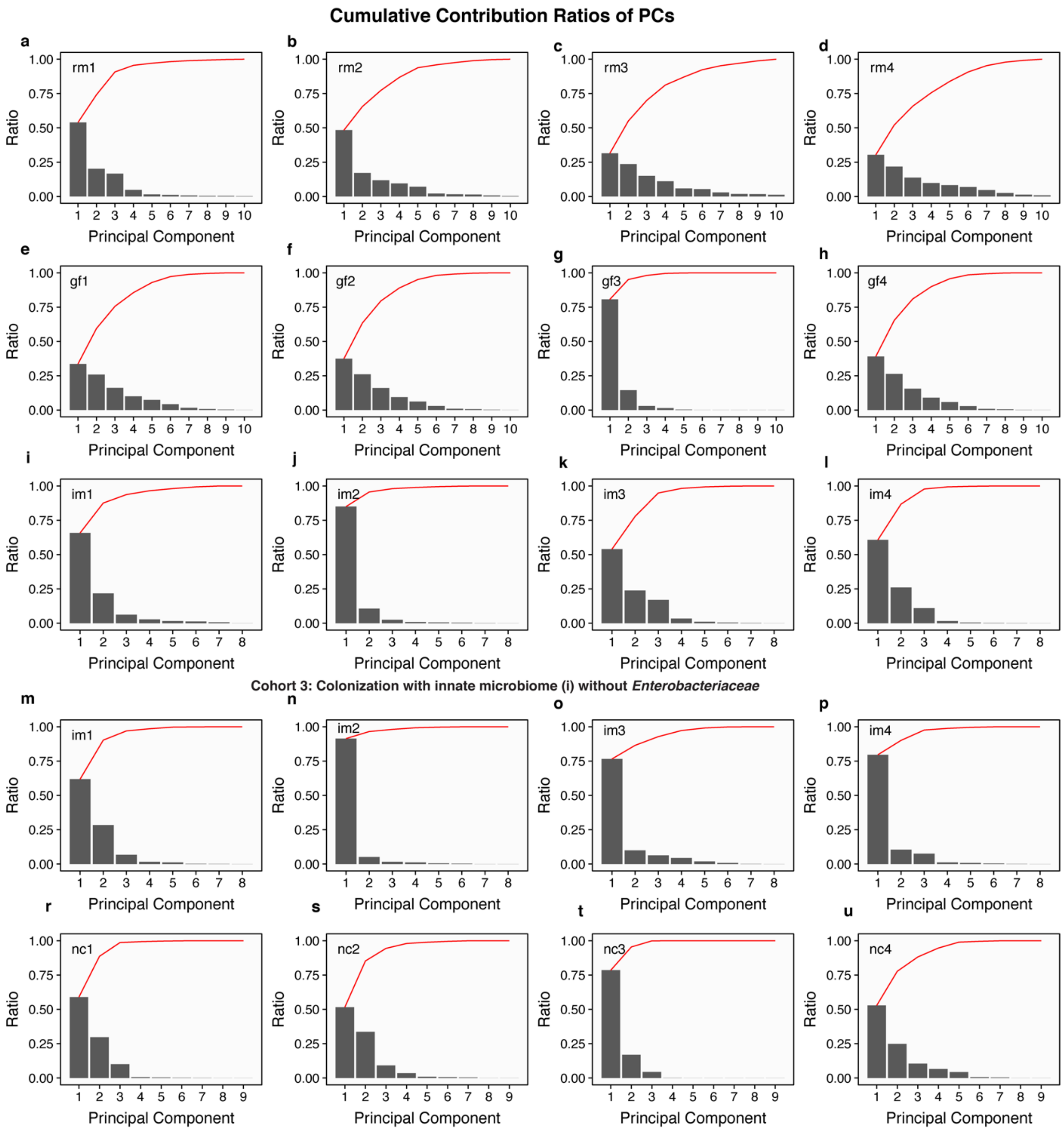

Supplementary Fig. 9 Principal components of the eigenvalue matrix of over the entire time-series.

a-d, Variance explained by each principal component (black) in the mice of the rm cohort. Red curve shows the cumulative variance explained. **e-p**, Principal components for the gf (panels e-h), im (panels i-l), im without gavaged *E. coli* (m-p) and nc (panels r-u) cohorts.

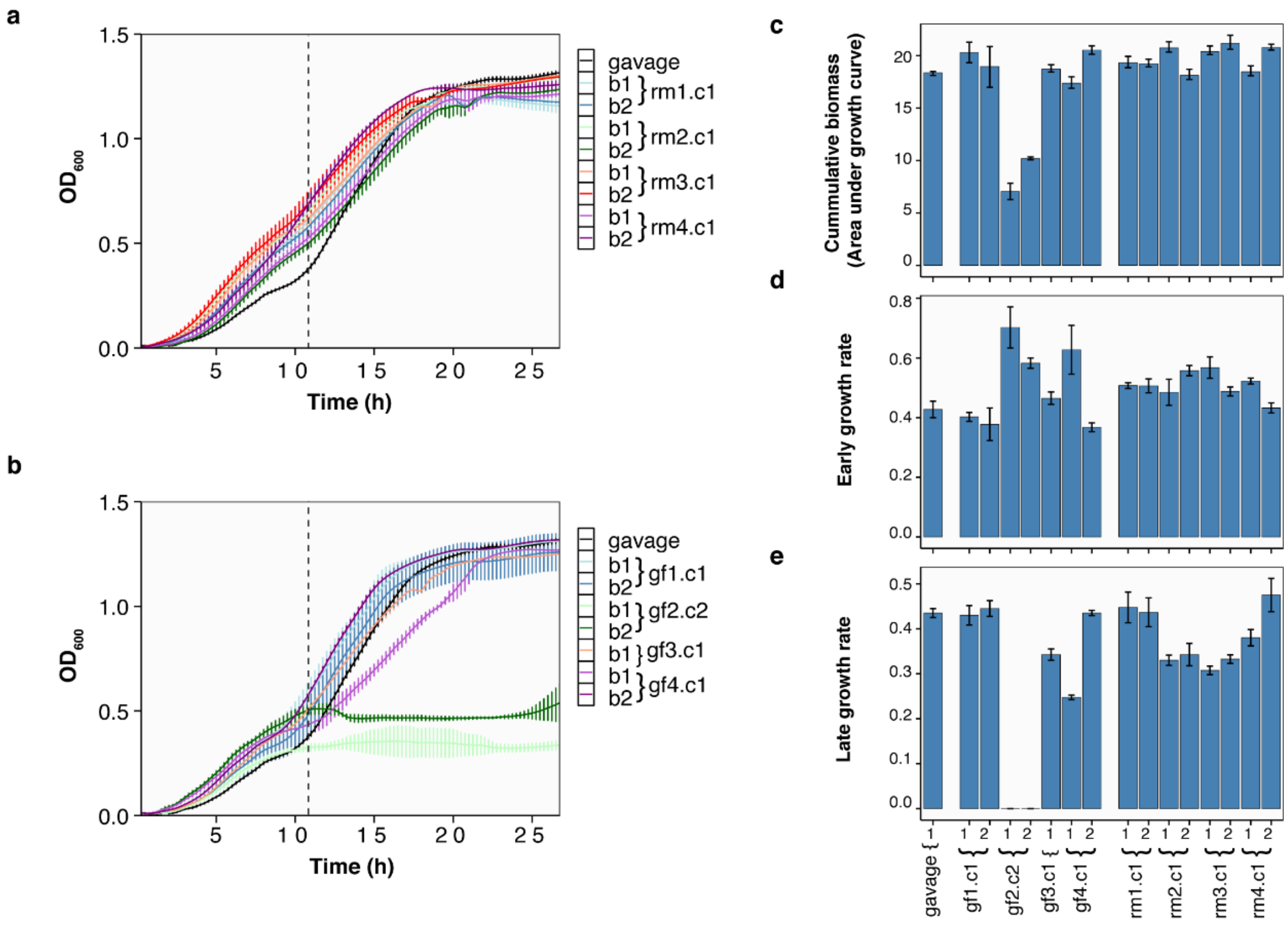

Supplementary Fig. 10 Growth rates of clones screened for whole-genome sequencing.

a, Growth curves in minimal media of isolated colonies from the rm cohort, all coming from the C1 cluster clone. The identifier b1 and b2 refer to isolated clones with different chromosomal barcodes. **b**, Growth curves for isolated colonies from the gf cohort, all clones are from clonal cluster C1, except mouse gf2. **c**, Fitness reported as cumulative biomass estimated as the area under the growth curve. **d,e**, Due to the bi-phasic nature of the growth curves, presumably due to initial adaptation to growth media, we calculated two growth rates, one corresponding to an early phase (before 12 hours) (panel d) and a late phase (after 12 hours) (panel e).

Supplementary Text

A. Sensitivity of dominant clonal lineage clusters on experimental parameters

A.1. Effect of number of input genomes to PCR amplification of barcode region

To determine the sensitivity of clonal lineage clustering pipeline, we first performed evolutionary simulations to arrive at a "noise-free" data of *E. coli* barcode dynamics, which will serve as "ground truth" for modeling the effect of various experimental parameters. We explicitly do not ecological effects (interactions with other species) and population structuring since we wanted to determine the effects of experimental parameters, outside of these biological factors, and would require further assumptions and parameters. Briefly, we performed Wright-Fisher simulation under constant population size (N_e = 10^9) using SodaPop[3], and with *de novo* mutation and standing genetic variation . We ran this simulation for 400 generations (corresponding to ~16 days, assuming a doubling time of 66-76/min[4]. The simulation used the experimental barcode distribution of the gavage sample.

To determine the effect of how many unique genomes are assayed in the NGS, we progressively performed random down-sampling on the simulation data. The down-sampling models the effect of potentially insufficient number of input cells during gDNA extraction or efficiency of genomic extraction itself, or the concentration of input gDNA for the NGS library preparation. From our "ground-truth" simulation with 10^9 cells over 400 generations (~2 weeks), we randomly chose 6 time-points, where we reduced the number of barcodes several orders of magnitude (downsampling factor γ =1/10, 1/10^2, ..., 1/10^6), such that the number of unique genomes ranged from 10^9 to 10^3. The down-sampling is performed as a binomial process, where a barcode's frequency f is reduced by an amount $\Delta f = Binomial(f, \gamma)$. We applied the down-sampling on randomly chosen 6 timepoints, as opposed to all time-points, to determine if non-uniform between time-points would introduce artifactual oscillations in the barcode dynamics. See Supplementary Fig. 11a for the resulting barcode dynamics. We applied our lineage clustering approach to the down-sampled data, resulting the identification of clonal lineages C1, C2, and C3. This exercise of down-sampling and lineage clustering was performed 100 times, the average lineage cluster clones are shown in Supplementary Fig. 11b.

To determine the effect of clustering misassignment due to down-sampling, we calculated
we calculated the overlap in barcode composition in the lineages of down-sampled data vs. no down-sampling for the dominant lineage clusters. We use the Simpson similarity between two sets ("lineage cluster") A and B, $OC(A,B) = \frac{|A \cap B|}{\min(|A|,|B|)}$, where a value close to 1 indicates a high number of common elements and a value near 0 indicates little overlap. Additionally, to determine the change in the lineage cluster dynamics induced by down sampling, we also determined the average "distance" in the timeseries of the corresponding clones as $d_{A,B} = \frac{1}{n}\sum_{i=1}^{n}\sqrt{(f_{A,i} - f_{B,i})^2}$, where $f_{A,i}$ and $f_{B,i}$ are the log10-frequency the clonal lineage abundance at the timepoint i averaged over all timepoints n. We perform 100 down-sampling simulations, and for each we calculated the "distance" and overlap of the resulting cluster clones to the "ground-truth". The mean and the standard deviation of the LCC's over the 100 simulations are shown in Supplementary Fig. 11b.

Expectedly, the clonal cluster with the lowest frequency is most sensitive to down-sampling. Altogether, the down-sampling exercise demonstrates that the quality of clonal cluster and the error in its average abundance frequency degrade when the input number of genomes is approximately 10^5. We note that our clonal cluster analysis is focused on the barcode lineages that are persistent and present at least

75% of the time period, which in our data includes the top ~5% of the barcodes. For other studies that use chromosomal barcodes to quantify the distribution of fitness effects (DFE) of mutations, as was done in reference[5], the analysis would require accuracy across all barcodes.

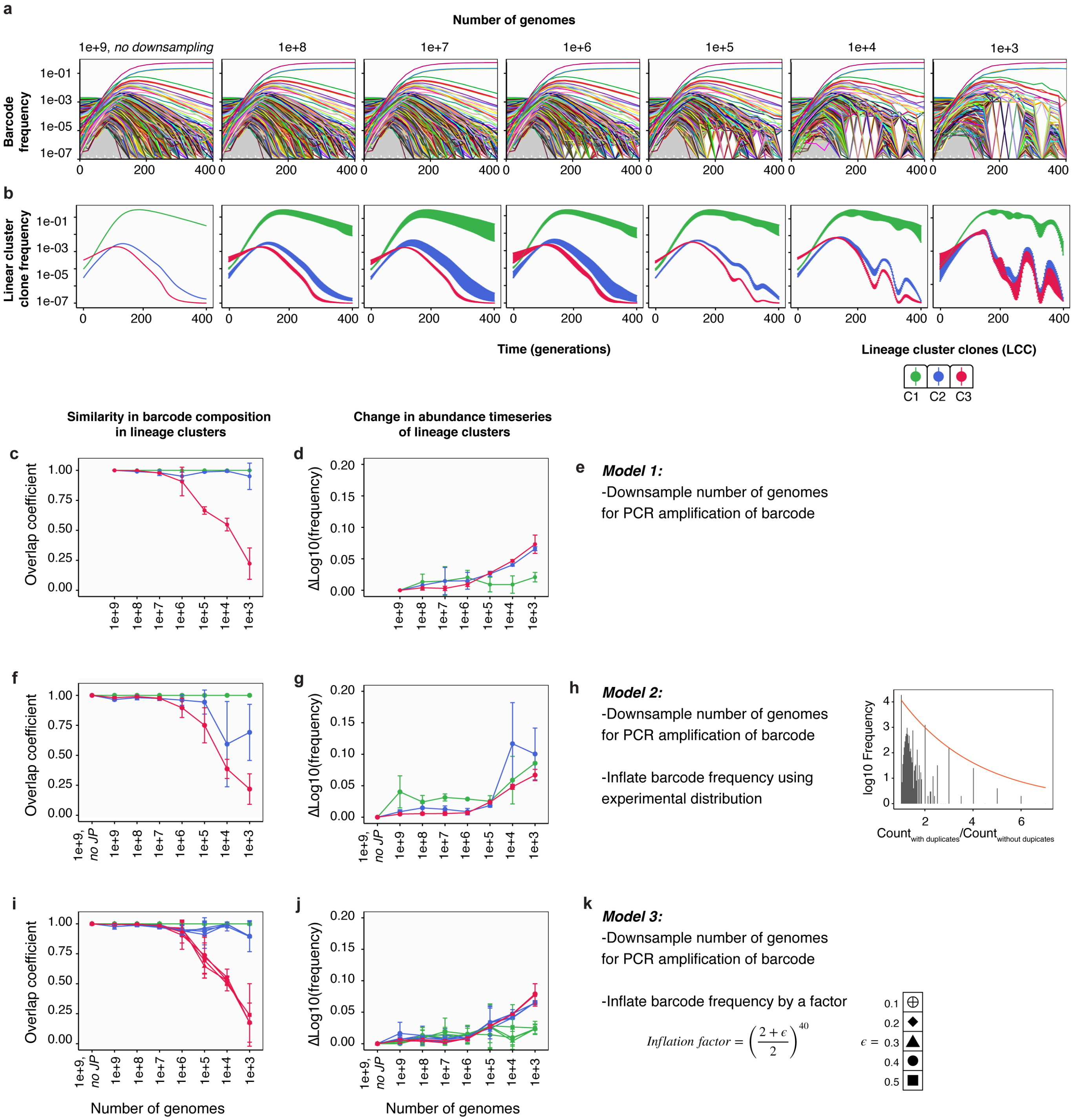

Supplementary Fig. 11 Effect of genome quantity and PCR bias on lineage cluster clones (skewed initial distribution)

a, Simulation of barcode evolution using the initial (t=0) experimental gavage distribution (leftmost panel). The number of genomes was sequentially downsampled by an order of magnitude until reaching 1e+3. **b,** Lineage cluster clones identified by applying the clustering pipeline to the data in panel a. The

average (solid lines) and error bars for the three dominant lineage clusters (C1, C2, and C3) were calculated from 100 independent down-sampling simulations. **c, d, e,** overlap in barcode compositions (panel c) and differences in the time series (panel d) between corresponding dominant lineage cluster clones with and without down sampling (illustrated in Model 1, panel e). The integrity of low-frequency clonal clusters deteriorates when the number of genomes is approximately 1e+5. Refer to the Supplementary Information text for more details. **f, g, h,** Additional stochasticity, which could potentially arise from variations in the amplification of different barcodes, was introduced into the down sampled data (Model 2). The increase in frequency is based on experimentally derived variations in barcodes using Unique Molecular Identifiers (UMIs). In panel h, the bars represent the experimental data, and the red line is an exponential fit (mean = 1.3 and sd=0.3) **i, j, k,** In Model 3 (panel k), we assume that PCR bias results from some barcodes increasing in frequency at a faster rate, approximately $(2+\epsilon)^n$, compared to the baseline rate, 2^n, over 40 PCR cycles. Similar to Model 2, in Model 3, the integrity of low-frequency clonal clusters deteriorates when the number of genomes is approximately 1e+6.

A.2. Effect of PCR jackpotting

PCR "jackpotting" refers to the stochastic nature of PCR, meaning that some molecules are amplified earlier than others. This leads to exponential amplification in subsequent rounds of PCR, distorting the frequency of the barcode distribution in the sample. We model this effect in two ways. We utilize an experimental distribution of PCR variability that employed UMI indices (Supplementary Fig. 11h, Model 2). Briefly, an in-house biological sample containing ~4000 DNA barcodes (from another project) was amplified for four cycles in the first PCR to add UMI. Then, the UMI-labeled products were amplified for 24 cycles to add Illumina P5/P6 primers in duplicates. The variation in read count (per UMI) in the samples, Supplementary Fig. 11h, was used as the distribution for the PCR variation effect, which includes jackpotting. From the experiment, we also found that ~25% of the barcodes are sensitive to such variation.

We randomly selected a barcode in the simulated data and altered its abundance by a factor randomly drawn from the distribution in Supplementary Fig. 11h. This was applied to all down sampled data in Supplementary Fig. 11a. The effect on clonal cluster time series and barcode composition is shown in Supplementary Fig. 11f,g. The simulation suggests that PCR jackpotting has a weaker effect than the number of genomic input to the PCR amplification. With both down-sampling and "jackpot" model 2, the clonal cluster quality for low-frequency variant is stable if the number of genomic input is ~1e+6.

To develop a more general model of PCR jackpotting, we postulated that jackpotting introduces an amplification of some barcodes faster than the background (Supplementary Fig. 11k). We formally modeled this inflation as $(2+\epsilon)^n/2^n$, where ϵ is in {0.1, 0.2, 0.3, 0.4, 0.5} and n is the number of cycles (set to 40, as was done in the experiment). In the worst-case scenario of our model, the inflation due to jackpot PCR is $(2+0.5)^{40}/2^{40}$, which is approximately 7E+3. In this scenario, the largest source of variation in the model is still the input genomic DNA (gDNA) (Supplementary Fig. 11i,j).

Taken together, considering the possible sources of error from down sampling, the skewness of the starting input frequency, and PCR jackpotting, maintaining the input number of cells at ~10^6 or above provides sufficient accuracy for the clonal cluster analysis.

A.3. Effect of skewedness of barcode frequency at time t=0

We have already accounted for the experimental skewness in barcode distribution as shown in Supplementary Fig. 11. However, for completeness, we investigated the potential improvements in the robustness of lineage clustering that could be achieved with a more uniform barcode distribution at t=0. To this end, we

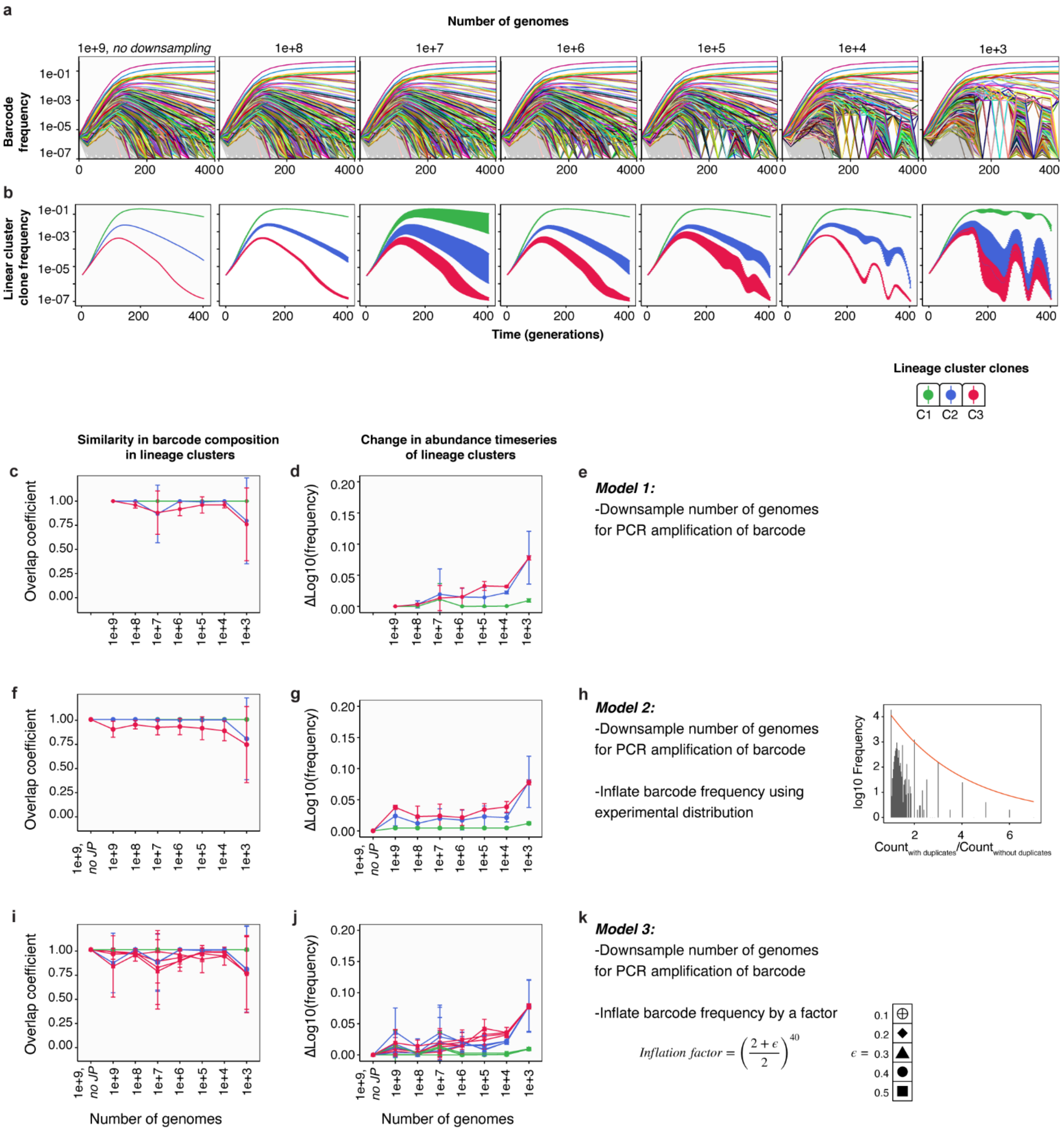

Supplementary Fig. 12 Effect of genome quantity and PCR bias on lineage cluster clones (uniform initial distribution)

a, Simulation of barcode evolution using uniform abundances of barcode at t=0 (leftmost panel). The number of genomes was sequentially down sampled by an order of magnitude until reaching 1e+3. **b,** Lineage cluster clones identified by applying the clustering pipeline to the data in panel a. The average (solid lines) and error bars were calculated from 1,000 independent down sampling simulations. **c, d, e,** overlap in barcode compositions (panel c) and differences in the time series (panel d) between corresponding dominant lineage cluster clones with and without down sampling (illustrated in Model 1, panel e). Low-frequency clonal clusters deteriorates when the number of genomes is approximately 1e+4. **f, g, h,** Additional stochasticity, which could potentially arise from variations in the amplification of different barcodes, was introduced into the down sampled data (Model 2). The increase in frequency is based on experimentally derived variations in barcodes using Unique Molecular Identifiers (UMIs). In panel h, the bars represent the experimental data, and the red line is an exponential fit (mean = 1.3 and sd=0.3). **i, j, k,** In Model 3 (panel k), we assume that PCR bias results from some barcodes increasing in frequency at a faster rate, approximately $(2+\epsilon)^n$, compared to the baseline rate, 2^n, over 40 PCR cycles. Similar to Model 2, in Model 3, the integrity of low-frequency clonal clusters deteriorates when the number of genomes is approximately ~1e+5.

performed a set of Wright-Fisher simulations with barcodes initialized at equal abundances (Supplementary Fig. 12a), while keeping all other simulation parameters consistent with those in Supplementary Fig. 11a. We then applied down sampling to assess the clustering sensitivity to the number of input genomic DNA molecules (Supplementary Fig. 12b). As anticipated, a perfectly uniform starting distribution of barcodes proved to be more resilient to the experimental effects of down sampling (Supplementary Fig. 12c-e). With a uniform distribution, we achieved sufficient accuracy in clonal cluster analysis with approximately 1e+4 input cells. The application of the two jackpotting models reduced this threshold to approximately 1e+5 input cells (Supplementary Fig. 12f-k).

Overall, this suggests that minimizing barcode skewness, when feasible, can enhance the robustness of the analysis against experimental noise from genomic extraction or PCR bias. Biologically, it implies that lineage clustering could be feasible in scenarios with lower bacterial loads that result from colonization.

A.4. Plasmid vs. chromosomal barcoding

Lineage tracking by DNA barcoding during gut colonization can also be achieved via other methods. For example, Vasquez et al. utilized ~200 barcodes (96 in each of two replicates) in plasmids to explore colonization lineage dynamics in germ-free mice. This approach followed earlier work by Cira et al. [6] who also used plasmid barcoding. An advantage of barcoding via plasmids, especially when the diversity is small (as with the ~200 barcodes), is that the barcoded plasmid can be introduced into cells individually. These cells can then be mixed at "equal concentrations" to create a uniform initial distribution of barcodes in the population, potentially guarding against sampling bias (see Supplementary Notes section A). Controlling the skewedness of barcoded cells is more challenging using the two-plasmid system of chromosomal barcoding that we use. Conversely, chromosomal barcoding offers advantages in ensuring stronger lineage assignment between parent and daughter cells, as there is a reduced likelihood of barcode transfer to other cells via conjugation (bacterial conjugation rate within a species is approximately 5×10^{-10} (cells/mL · min)). Although plasmid loss rates can vary significantly, from as low as 1.5×10^{-3} per cell to as high as 1–5% per cell per generation [7-10], there is a lower risk of the barcode being lost if it resides on the chromosome, as opposed to a plasmid. Altogether, the choice between

plasmid-based or chromosomal barcoding depends on available lab resources and the specific requirements of the desired application.

B. DCM analysis on generalized Lotka-Volterra models and the effect of time-series sampling accuracy

B.1. Correspondence with generalized Lotka-Volterra

In the case of the gLV, Eq. 2 in the main text is

$$\dot{z}_i = z_i\phi_i = z_i(\vec{r} + A\vec{z}) \qquad \text{(S1)}$$

where $\vec{\phi} = \vec{r} + A\vec{z}$. The vector $\vec{r}$ is the growth rates and the matrix $A = [a_{ij}]$ is the interaction strength matrix, denoting the impact of abundance of species *j*, z_i, on the growth rate of species *i*, r_i. The off-diagonal elements of the Jacobian of the gLV are:

$$J_{ij} = \frac{d\phi_i}{dz_j} = a_{ij}z_i \qquad \text{(S2)}$$

and the diagonal elements are:

$$J_{ii} = \frac{d\phi_i}{dz_i} = \left(r_i + \sum_j a_{ij}z_j\right) + a_{ii}z_i \qquad \text{(S3)}$$

At equilibrium, assuming that the abundance vector is z^*, $\frac{dz_i}{dt} = 0$, which from Eq. S1 also implies that $\vec{\phi} = 0$ and $\left(r_i + \sum_j a_{ij}z_j\right) = 0$. Thus, under equilibrium, $J_{ij} = a_{ij}z_i^*$, showing that in the gLV, that the elements of the interaction strength matrix A are correlated to the elements of the community matrix *J* or the Jacobian, up to a factor that is determined by the equilibrium abundance (which is positive $z_i^* > 0$ for any persistent species). There are fundamental mathematical considerations about the existence of local or global stable equilibrium for Eq. S1, which is an extensive field of research in itself [11, 12].

B.2 Capturing community shifts without prior knowledge using gLV

We benchmarked our results against standard gLV models with constant interaction matrices. Specifically, we used web-gLV (Kuntal et al., *Frontiers in Microbiology*, 2019[13]) that estimates the interaction matrix from time-series data. Similar to DCM, we did not inform web-gLV that there is a community shift at t=20. Supplementary Fig. 1g-h shows that the estimated community matrix by web-gLV over the entire period does not correlate with either A1 or with A2, as expected since the community shift is not in the framework of constant interaction gLV. Of course, gLV could be forced to model a shift in the community interaction, but this information will need to be known up front to partition the abundance time-series into segments where constant community matrix applies.

These results show DCM captures the shift dynamics within a constant gLV matrix (such as approach to equilibrium), as well as shifts in the community matrix. Importantly, the DCM does not need to be informed *a priori* of this community shift, as one would in a gLV constant matrix model.

B.3. Effect of time sampling on community abundances

Shown in Supplementary Fig. 13a (leftmost panel) is the stable-oscillatory co-existence among three species within an gLV system. Increasing the sampling timestep, Δt, as presented in Supplementary Fig. 11a, could result in a less accurate representation of the interspecies interactions. Analogous to the approach taken for the microbial gut time-series, we estimated the Jacobian as the covariance of species i's time derivatives and species j's abundance time-series over the interval τ, defined as $J_\tau = \langle \mathrm{cov}(\dot{z}_i, z_j) \rangle$. For Supplementary Fig. 13b, we selected τ to be 1. Indeed, there is a correlation between the elements of the Interaction Strength and Community matrices (see Supplementary Fig. 13c for Δt=1). The correspondence between J_τ and A hinges on the frequency at which the species' abundances are sampled over time. Less frequent sampling of the abundance time-series implies a reduced capacity to capture the interactions between species (as shown in Supplementary Fig. 11d).

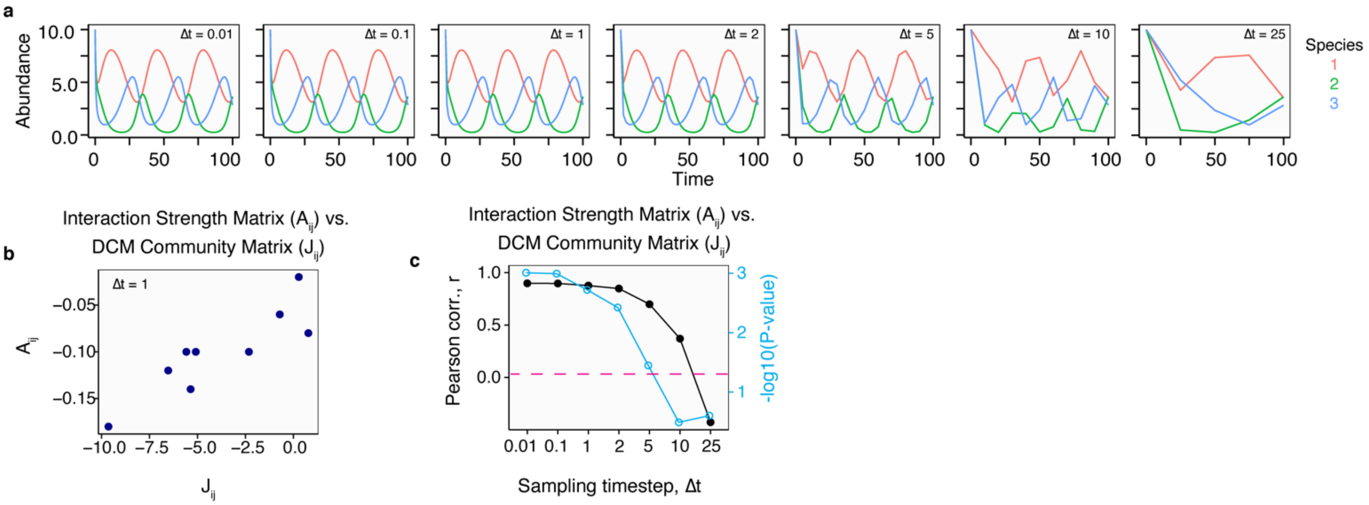

Supplementary Fig. 13 Three-species Lotka-Volterra system with stable oscillatory coexistence.

a Abundance timeseries (panel a) of the 3-species defined by the interaction strength matrix A. All three species were initialized to abundance equal to 10. The panels correspond to different sampling timestep Δt. Time and abundances are in arbitrary units. **b**, Element-wise comparison of the Interaction Strength and Community matrices estimated by DCM for $\Delta t = 1$. **c,** The strength and significance of correlation between Interaction Strength and estimated Community matrices drop with increasing sampling timestep.

C. Co-clustering between *E. coli* clonal cluster lineages and the community dynamics

Typical approaches to understanding species interactions rely on co-occurrence measures, interpreting species correlation data as signs of mutual dependency[14, 15]. Yet, numerous studies indicate that communities exhibit complex dynamics—such as multiple phases and oscillations that are driven by the interplay between community members and environmental factors[16, 17]. Thus, these community interactions can create local and transient stretching or lags on population dynamics. Therefore, to detect the information between clonal clusters and community members, we adopted a strategy that uses normalized cross-correlation measures to consider the shapes of time series[18]. This clustering approach is able to characterize community structure based on the temporal behavior of species or clonal clusters rather than fitting a model or checking pairwise correlations (see Methods).

C.1. Assessing the biological significance of the co-clustering of clonal clusters and community dynamics

To validate that our co-clustering method between the community and clonal dynamics is significant, we calculated a metric called "mixing index". The underlying rationale was that if indeed, clustering of an *E. coli* clonal lineage with a bacterial family is biologically meaningful, then this clustering should be strongest when both clonal lineage dynamics and 16S come from the same mice or same cohort. To assess the mixing index, we collect clone-clone *cophenetic* distances (c) and clone-species *cophenetic* distances (m) from their respective co-clustering. (Cophenetic distance is the distance between two leaves of a hierarchical tree and is defined as the height of the closest node that leads to both leaves). Then the distance between the empirical cumulative distributions of c and m, denoted as $F(c)$ and $F(m)$ respectively, is quantified as

$$D_{c,m} = 1 - (max|(F(c) - F(m)|) \quad \text{(S4)}$$

Higher values of the mixing index imply that clonal clusters and families are more likely to be adjacent leaves in the co-clustering three than clonal clusters amongst themselves. As an illustration, we show in Supplementary Fig. 14 ,the mixing indices for trees where clonal clusters and families are fully mixed, partly mixed, and fully unmixed. We applied the mixing index to co-clustering trees arising from different pairs of clonal lineages (im, rm or gf) and bacterial families (im, rm, or nc). Furthermore, to determine the robustness of the mixing with respect to the method for determining the dominant clonal lineages (section v), we evaluated the mixing index different cut-off thresholds for lineage clustering (Supplementary Fig. 6). The mixing index values are shown as violin plots in Fig. 4b. We found that the mixing index is largest when the clonal lineages and bacterial families come from the same mouse cohort. The statistical significance between the mixing indexes was quantified by a two-tailed t-test.

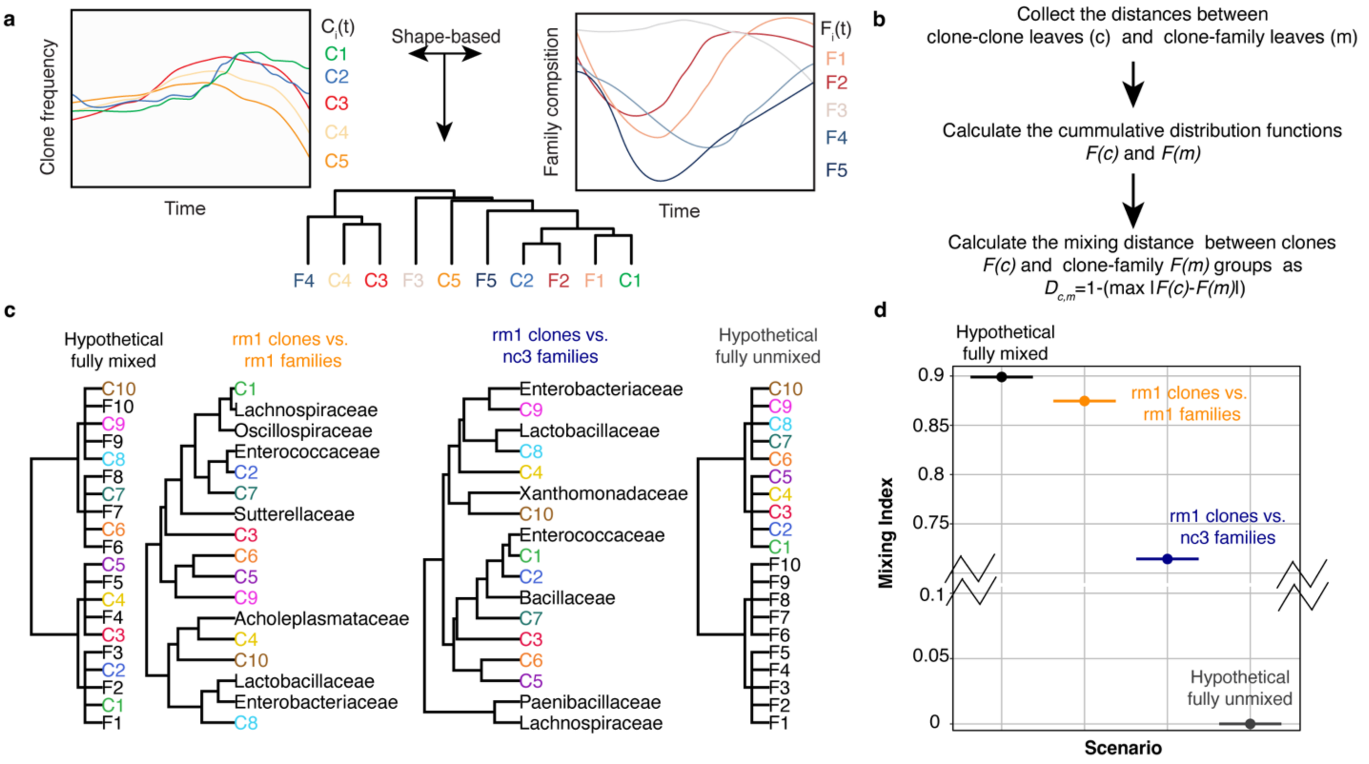

Supplementary Fig. 14 Schema for shaped-based co-clustering between *E. coli* clone and community dynamics.

a, Schema for co-clustering of the clonal lineage and family composition time series using a shape-based distance metric. **b**, Schema for calculating the mixing index from a given hierarchical clustering tree. We collect clone-clone *cophenetic* distances (c) and clone-species *cophenetic* distances (m). Then the distance between the empirical cumulative distributions of c and m, denoted as $F(c)$ and $F(m)$ respectively, is quantified as $D_{c,m} = 1 - (max|(F(c) - F(m)|)$. Higher values of the mixing index imply that clonal clusters and families are more likely to be adjacent leaves in the co-clustering tree than clonal clusters amongst themselves. **c-d**, Illustrative examples (panel c) of different extents of co-clustering between clonal clusters and families and their corresponding mixing indices (panel d).

C.2. Robustness to E. coli clonal cluster lineages and the community dynamics to choice of distance and clustering methods

To determine if the co-clustering of community and clonal dynamics is robust to our choice of clustering methods, we conducted additional analyses to explore the associations between barcode clusters and microbial families using correlation-based methods with time-lag parameters. Specifically, we employed Lag-Penalized Weighted Correlation (LPWC; https://gitter-lab.github.io/LPWC/articles/LPWC.html and Chandereng & Gitter. BMC Bioinformatics 21, 21 (2020) [19]), which groups pairs of time series that exhibit closely related behaviors over time, even if their timing is not perfectly synchronized (presence of lags). LPWC aligns time series profiles to identify common temporal patterns, penalizing the correlations of lagged profiles based on the length of the introduced temporal lags using a Gaussian kernel.

We compared the dendrograms generated from shape-based distance (SBD) clustering and LPWC (Supplementary Fig. 15). The dendrograms from both observed that they share similar structures, as quantified by cophenetic correlation (p-values are indicated in Supplementary Fig. 15). This suggests that, even with lag-free methodologies, the dendrograms are closely aligned and shape-based clustering can indeed capture lag-penalized temporal variations effectively. Altogether, by incorporating both shape-based and correlation-based methods with time lags, we provide a more robust representation of how barcode clusters and microbial families interact over time.

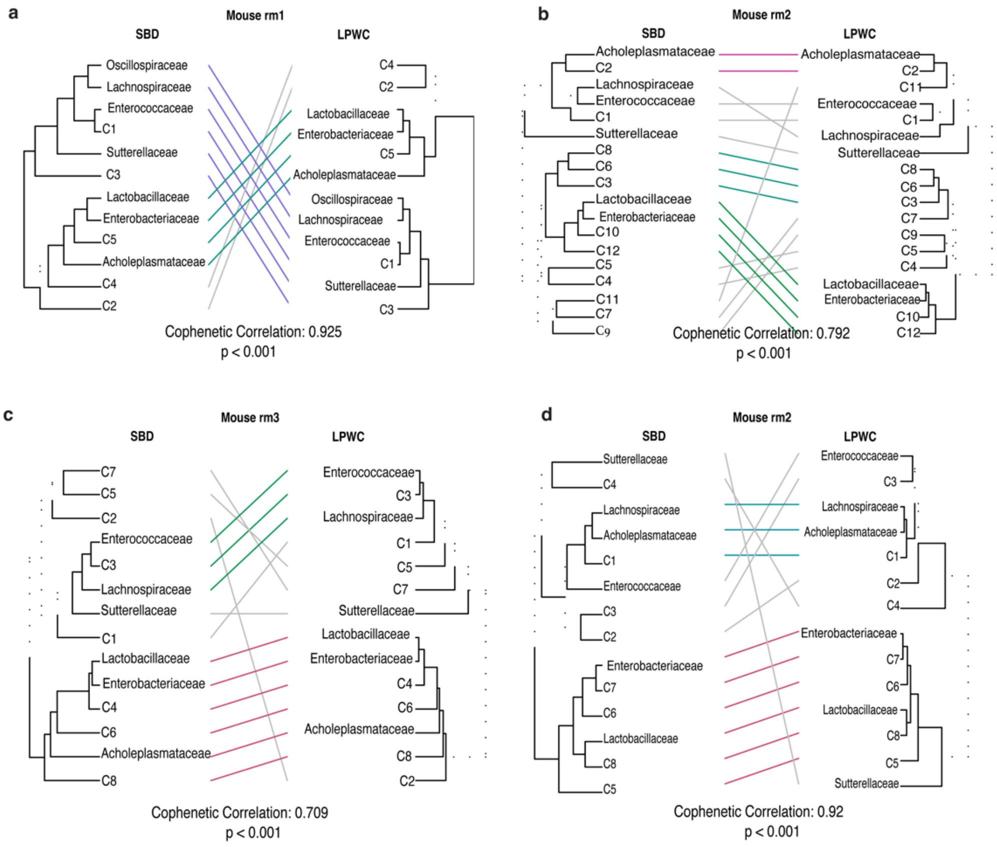

Supplementary Fig. 15 Comparison of rm cohort co-clustering using shape-based distance (SBD) with lag-penalized weighted clustering (LPWC).

a-d, first dendrogram created with shape-based distance; second dendrogram generated using LPWC. To assess the similarity between these clustering methods, we computed the cophenetic correlation coefficient, which quantifies how well each clustering method preserves pairwise dissimilarities between samples. The cophenetic correlation was greater than 0.7, for all samples indicating a significant correlation between the two clustering approaches. Colored lines, generated using the tanglegram function from the dendextend[20] R package, connect common branches between the two dendrograms, visually highlighting their structural similarity.

References:

1. Bucci, V. *et al.* in Genome Biology, Vol. 17 1-17 (Genome Biology, 2016).
2. Dickerson, R.E. *et al.* The Anatomy of a-DNA, B-DNA, and Z-DNA. *Science* **216**, 475-485 (1982).
3. Gauthier, L., Di Franco, R. & Serohijos, A.W.R. SodaPop: a forward simulation suite for the evolutionary dynamics of asexual populations on protein fitness landscapes. *Bioinformatics* **35**, 4053-4062 (2019).
4. Barroso-Batista, J., Demengeot, J. & Gordo, I. Adaptive immunity increases the pace and predictability of evolutionary change in commensal gut bacteria. *Nat Commun* **6**, 8945 (2015).
5. Levy, S.F. *et al.* Quantitative evolutionary dynamics using high-resolution lineage tracking. *Nature* **519**, 181-186 (2015).

6. Cira, N.J., Pearce, M.T. & Quake, S.R. Neutral and selective dynamics in a synthetic microbial community. *P Natl Acad Sci USA* **115**, E9842-E9848 (2018).
7. Boe, L., Gerdes, K. & Molin, S. Effects of Genes Exerting Growth-Inhibition and Plasmid Stability on Plasmid Maintenance. *Journal of Bacteriology* **169**, 4646-4650 (1987).
8. Boe, L. & Rasmussen, K.V. Suggestions as to quantitative measurements of plasmid loss. *Plasmid* **36**, 153-159 (1996).
9. Dam, M. & Gerdes, K. Partitioning of Plasmid R1 - 10 Direct Repeats Flanking the Para Promoter Constitute a Centromere-Like Partition Site Parc, That Expresses Incompatibility. *Journal of Molecular Biology* **236**, 1289-1298 (1994).
10. Nordstrom, K. & Aagaardhansen, H. Maintenance of Bacterial Plasmids - Comparison of Theoretical Calculations and Experiments with Plasmid-R1 in Escherichia-Coli. *Mol Gen Genet* **197**, 1-7 (1984).
11. Dynamical Systems and Lotka–Volterra Equations, in *Evolutionary Games and Population Dynamics*. (eds. J. Hofbauer & K. Sigmund) 1-2 (Cambridge University Press, Cambridge; 1998).
12. Hui, C. & Richardson, D.M. How to Invade an Ecological Network. *Trends Ecol Evol* **34**, 121-131 (2019).
13. Kuntal, B.K., Gadgil, C. & Mande, S.S. Web-gLV: a web based platform for Lotka-Volterra based modeling and simulation of microbial populations. *Frontiers in microbiology* **10**, 288 (2019).
14. Faust, K. & Raes, J. Microbial interactions: from networks to models. *Nat Rev Microbiol* **10**, 538-550 (2012).
15. Friedman, J. & Alm, E.J. Inferring Correlation Networks from Genomic Survey Data. *Plos Computational Biology* **8** (2012).
16. Fisher, L. Critical Transitions in Nature and Society. *Am J Psychol* **124**, 365-367 (2011).
17. Scheffer, M., Carpenter, S., Foley, J.A., Folke, C. & Walker, B. Catastrophic shifts in ecosystems. *Nature* **413**, 591-596 (2001).
18. Paparrizos, J. & Gravano, L. k-Shape: Efficient and Accurate Clustering of Time Series. *SIGMOD Rec.*, 1855-1870 (2015).
19. Chandereng, T. & Gitter, A. Lag penalized weighted correlation for time series clustering. *BMC Bioinformatics* **21**, 21 (2020).
20. Galili, T. dendextend: an R package for visualizing, adjusting and comparing trees of hierarchical clustering. *Bioinformatics* **31**, 3718-3720 (2015).
