# Supplementary material for: Quantifying the intra- and inter-species community interactions in microbiomes by dynamic covariance mapping
Source: Nat Commun. 2025 Jul 9;16:6314. doi: 10.1038/s41467-025-61368-y (PMC12238654; doi:10.1038/s41467-025-61368-y)
Supplement: Supplementary file 2 — Description of Additional Supplementary Files [file 41467_2025_61368_MOESM2_ESM.docx]

**Description of Additional Supplementary Files**

**Filename: Supplementary Movie 1.**

**Description:** DCM applied to the 5-species gLV system.

**Filename: Supplementary Movie 2.**

**Description:** DCM applied to the mouse gut community perturbed by pathogenic bacteria.

**Filename: Supplementary Movie 3.**

**Description:** DCM applied to the mouse 1 of rm cohort

**Filename: Supplementary Movie 4.**

**Description:** DCM applied to the mouse 2 of rm cohort

**Filename: Supplementary Movie 5.**

**Description:** DCM applied to the mouse 3 of rm cohort

**Filename: Supplementary Movie 6.**

**Description:** DCM applied to the mouse 4 of rm cohort

**Filename: Supplementary Movie 7.**

**Description:** DCM applied to the mouse 1 of gf cohort

**Filename: Supplementary Movie 8.**

**Description:** DCM applied to the mouse 2 of gf cohort

**Filename: Supplementary Movie 9.**

**Description:** DCM applied to the mouse 3 of gf cohort

**Filename: Supplementary Movie 10.**

**Description:** DCM applied to the mouse 4 of gf cohort

**Filename: Supplementary Movie 11.**

**Description:** DCM applied to the mouse 1 of im cohort

**Filename: Supplementary Movie 12.**

**Description:** DCM applied to the mouse 2 of im cohort

**Filename: Supplementary Movie 13.**

**Description:** DCM applied to the mouse 3 of im cohort

**Filename: Supplementary Movie 14.**

**Description:** DCM applied to the mouse 4 of im cohort

**Filename: Supplementary Movie 15.**

**Description:** DCM applied to the mouse 1 of im cohort, but with *Enterobacteriaceae* excluded.

**Filename: Supplementary Movie 16.**

**Description:** DCM applied to the mouse 2 of im cohort, but with *Enterobacteriaceae* excluded.

**Filename: Supplementary Movie 17.**

**Description:** DCM applied to the mouse 3 of im cohort, but with *Enterobacteriaceae* excluded.

**Filename: Supplementary Movie 18.**

**Description** DCM applied to the mouse 4 of im cohort, but with *Enterobacteriaceae* excluded.

**Filename: Supplementary Movie 19.**

**Description** DCM applied to the mouse 1 of nc cohort

**Filename: Supplementary Movie 20.**

**Description:** DCM applied to the mouse 2 of nc cohort

**Filename: Supplementary Movie 21.**

**Description:** DCM applied to the mouse 3 of nc cohort

**Filename: Supplementary Movie 22.**

**Description:** DCM applied to the mouse 4 of nc cohort

**Filename: Supplementary Data 1**

**Description:** Barcodes of screened colonies used for whole-genome sequencing.

**Filename: Supplementary Data 2**

**Description:** Mutations identified from metagenome or single colony whole-genome sequencing.
