# Supplementary material for: Quantifying the intra- and inter-species community interactions in microbiomes by dynamic covariance mapping
Source: Nat Commun. 2025 Jul 9;16:6314. doi: 10.1038/s41467-025-61368-y (PMC12238654; doi:10.1038/s41467-025-61368-y)
Supplement: Supplementary file 26 — Reporting summary [file 41467_2025_61368_MOESM26_ESM.pdf]

nature portfolio

Corresponding author(s): *Double-anonymous peer review submissions: write DAPR and your manuscript number here instead of author names.*

Last updated by author(s): *YYYY-MM-DD*

Reporting Summary

Nature Portfolio wishes to improve the reproducibility of the work that we publish. This form provides structure for consistency and transparency in reporting. For further information on Nature Portfolio policies, see our Editorial Policies and the Editorial Policy Checklist.

Statistics

For all statistical analyses, confirm that the following items are present in the figure legend, table legend, main text, or Methods section.

n/a	Confirmed	
☐	☒	The exact sample size (*n*) for each experimental group/condition, given as a discrete number and unit of measurement
☐	☒	A statement on whether measurements were taken from distinct samples or whether the same sample was measured repeatedly
☐	☒	The statistical test(s) used AND whether they are one- or two-sided<br>*Only common tests should be described solely by name; describe more complex techniques in the Methods section.*
☐	☒	A description of all covariates tested
☐	☒	A description of any assumptions or corrections, such as tests of normality and adjustment for multiple comparisons
☐	☒	A full description of the statistical parameters including central tendency (e.g. means) or other basic estimates (e.g. regression coefficient) AND variation (e.g. standard deviation) or associated estimates of uncertainty (e.g. confidence intervals)
☐	☒	For null hypothesis testing, the test statistic (e.g. *F*, *t*, *r*) with confidence intervals, effect sizes, degrees of freedom and *P* value noted<br>*Give P values as exact values whenever suitable.*
☒	☐	For Bayesian analysis, information on the choice of priors and Markov chain Monte Carlo settings
☐	☒	For hierarchical and complex designs, identification of the appropriate level for tests and full reporting of outcomes
☐	☒	Estimates of effect sizes (e.g. Cohen's *d*, Pearson's *r*), indicating how they were calculated

Our web collection on statistics for biologists contains articles on many of the points above.

Software and code

Policy information about availability of computer code

Data collection	Data collection are described in the methods section.
Data analysis	Data analysis and the codes are described in the methods section and posted in Github https://github.com/melisgncl/Intra--and-inter-species-interactions-drive-phases-of-invasion-in-gut-microbiota-

For manuscripts utilizing custom algorithms or software that are central to the research but not yet described in published literature, software must be made available to editors and reviewers. We strongly encourage code deposition in a community repository (e.g. GitHub). See the Nature Portfolio guidelines for submitting code & software for further information.

Data

Policy information about availability of data

All manuscripts must include a data availability statement. This statement should provide the following information, where applicable:

- Accession codes, unique identifiers, or web links for publicly available datasets
- A description of any restrictions on data availability
- For clinical datasets or third party data, please ensure that the statement adheres to our policy

Raw barcode sequencing data from this study have been deposited in the National Center for Biotechnology Information Sequence Read Archive. This includes BioProject accession number PRJNA1113167 for all 16S data, PRJNA1113343 for high-resolution barcode data from germ-free, reduced microbiota, and innate microbiota cohorts across different time points, and PRJNA1113345 for whole genome sequencing results of dominant clonal clusters lineages.

Research involving human participants, their data, or biological material

Policy information about studies with human participants or human data. See also policy information about sex, gender (identity/presentation), and sexual orientation and race, ethnicity and racism.

Reporting on sex and gender	The study does not involve human subjects.
Reporting on race, ethnicity, or other socially relevant groupings	The study does not involve human subjects.
Population characteristics	The study does not involve human subjects.
Recruitment	The study does not involve human subjects.
Ethics oversight	The study does not involve human subjects.

Note that full information on the approval of the study protocol must also be provided in the manuscript.

Field-specific reporting

Please select the one below that is the best fit for your research. If you are not sure, read the appropriate sections before making your selection.

[x] Life sciences [ ] Behavioural & social sciences [ ] Ecological, evolutionary & environmental sciences

For a reference copy of the document with all sections, see nature.com/documents/nr-reporting-summary-flat.pdf

Life sciences study design

All studies must disclose on these points even when the disclosure is negative.

Sample size	The details of the sample size and statistical analysis are described in the methods section. Specifically, there were 4 mice cohorts, each with at least 4 mice replicates. In each mice, we tracked ~500,000 single-cell DNA barcode for study the chromosomal dynamics.
Data exclusions	No data were excluded.
Replication	All replicates are reported.
Randomization	The mice allocated to each cohort were chosen randomly.
Blinding	Not applicable.

Reporting for specific materials, systems and methods

We require information from authors about some types of materials, experimental systems and methods used in many studies. Here, indicate whether each material, system or method listed is relevant to your study. If you are not sure if a list item applies to your research, read the appropriate section before selecting a response.

Materials & experimental systems

n/a	Involved in the study	
x		Antibodies
x		Eukaryotic cell lines
x		Palaeontology and archaeology
	x	Animals and other organisms
x		Clinical data
x		Dual use research of concern
x		Plants

Methods

n/a	Involved in the study	
x		ChIP-seq
x		Flow cytometry
x		MRI-based neuroimaging

Animals and other research organisms

Policy information about studies involving animals; ARRIVE guidelines recommended for reporting animal research, and Sex and Gender in Research

Laboratory animals	We used female C57BL6/N mice

Wild animals	The study does not involve wild animals.
Reporting on sex	All cohort mice were C57BL/6N (Taconic Biosciences), and only 12-week-old females were used. Animals were kept in filter-covered ventilated cages, maintained at 23°C with 40% humidity, under a 12-hour light/dark cycle.
Field-collected samples	Not applicable.
Ethics oversight	All experimental protocols on mice were approved by Université de Sherbrooke Ethics Committee for Animal Care and by University of Toronto's Animal Care Committee, both in accordance with guidelines established by the Canadian Council on Animal Care.

Note that full information on the approval of the study protocol must also be provided in the manuscript.

Plants

Seed stocks	The study does not involve plants.
Novel plant genotypes	The study does not involve plants.
Authentication	The study does not involve plants.
